# Spectral domain optical coherence tomography in children: normative data and biometric correlations

**DOI:** 10.1186/1471-2415-14-53

**Published:** 2014-04-22

**Authors:** Christiane Al-Haddad, Anita Barikian, Mahmoud Jaroudi, Vicky Massoud, Hani Tamim, Baha’ Noureddin

**Affiliations:** 1 Department of Ophthalmology, American University of Beirut, Beirut, Lebanon; 2 Department of Internal Medicine, American University of Beirut, Beirut, Lebanon

**Keywords:** Optical coherence tomography, Pediatric, Retinal nerve fiber layer, Macular thickness, Biometric correlations

## Abstract

**Background:**

The aim was to report normative values of retinal nerve fiber layer (RNFL) and macular parameters in children using spectral domain optical coherence tomography (OCT) and to perform correlations with age, refractive error and axial length.

**Methods:**

This was an observational cross-sectional study recruiting 113 healthy children aged 6 to 17 years with no ocular abnormality except refractive error. After a comprehensive eye examination and axial length measurement, RNFL and macular thickness measurements were performed using the Cirrus OCT machine. Main outcome measures were macular volume, macular thickness and RNFL thickness values as well as their correlations with age, refractive error and axial length. Right eyes of all subjects were selected for analysis.

**Results:**

One hundred and eight children were included in the study, 65 females and 43 males. Mean age was 10.7+/−3.1 years, average spherical equivalent refraction (SE) was −0.02+/−1.77(−4.25 to +5.00) diopters and average axial length was 23.5+/−1.0 (21.5 to 25.8)mm. Mean RNFL thickness was 95.6+/−8.7 μm, average macular thickness was 279.6+/−12.5 μm, central macular thickness was 249.1+/−20.2 μm, and mean macular volume was 10.1+/−0.5 mm^3^. Central macular thickness values were significantly higher in males (p < 0.001). RNFL measurements did not correlate with age but did show a positive correlation with SE. All macular parameters were consistently positively correlated with age and most of them were positively correlated with SE. When controlling for axial length, only the macular inner circle thickness was positively correlated with age.

**Conclusions:**

Using Cirrus OCT, normative RNFL and macular parameters in healthy children below 18 years of age were established; measurements varied by age and gender.

## Background

Optical Coherence Tomography (OCT) is a noninvasive, noncontact, transpupillary imaging method that performs objective high-resolution cross-sectional images of retinal tissue. The recently introduced spectral domain OCT (SD-OCT) provides measurements of the retinal nerve fiber layer (RNFL) and macula with greatly improved image acquisition speed and image resolution up to 5 μm [1]. This is particularly helpful when applying this technology in uncooperative children.

Several studies have proved the feasibility of OCT in the pediatric population [2-9]. Nevertheless, all OCT devices have an integrated normative database only for adult subjects 18 years of age and older. Earlier studies have reported normative values in children using the time domain OCT (TD-OCT) devices [3-11]; similar reports using SD-OCT are much less available. Although RNFL measurements taken from TD-OCT and SD-OCT are comparable, significant differences exist and values cannot be used interchangeably [12,13]. Comparing macular thickness between the two instruments is even more complicated, being dependant on pathology and location [14]. Measurement protocols vary even among different SD-OCT instruments [15]. Only a few studies in the literature aimed at reporting normative reference ranges using SD-OCT [16-21]. Normative measurements in children using Cirrus are least reported, especially with regards to macular parameters.

The clinical applications of SD-OCT are increasingly expanding [22]; normal reference values for RNFL and macular thickness are needed in the pediatric population where the software has no nomogram for comparison. The purpose of this study was to collect normative values for SD-OCT measurements of macular thickness and volume and peripapillary RNFL thickness in healthy eyes of normal children using the most recent commercially available Cirrus SD-OCT, and to study the effects of age, gender, axial length, and refractive error on these values.

## Methods

### Study population

This was a cross-sectional study of healthy white Middle Eastern children 6 to 17 years of age visiting the pediatric ophthalmology clinic at the American University of Beirut from November 2011 to September 2012. This group of children were referred because of failed school screening, visual behavior abnormalities noted by parents, positive family history of refractive errors, or referral from the pediatrician. The study was approved by the American University of Beirut Institutional Review Board. Written parental informed consent was obtained from parents or legal guardians; children and adolescent assent forms were also provided for children 7 years of age and older. One hundred and thirteen children and adolescents were enrolled prospectively and consecutively. Detailed demographic data were obtained during the clinic encounter. Included in the study were subjects with no ocular abnormality except refractive error less than 7.00 diopters (of hyperopic or myopic spherical equivalent), normal visual acuity (best corrected Snellen visual acuity of 20/20) and normal fundoscopy. Excluded were patients with a history of intraocular surgery, strabismus, anisometropia more than 1.50 diopters, amblyopia, retinal pathology, glaucoma, optic nerve cup to disc ratio >0.5 or asymmetry of >0.2 between fellow eyes. Patients with history of prematurity, neurologic, metabolic or other systemic diseases were also excluded.

### Ocular examination

All subjects received a comprehensive ophthalmologic examination by a pediatric ophthalmologist (CA). The visual acuity of each eye was recorded using the Snellen chart; intraocular pressure assessment, motility examination, stereoacuity testing, slit lamp exam, cycloplegic refraction, and dilated fundoscopy were performed. Pupils were dilated using cyclopentolate 1% eyedrops, instilled twice 10 minutes apart. Manual retinoscopy was performed 30 minutes after the last drop by a pediatric ophthalmologist (CA). This was also confirmed by automated refraction (Canon RK-F1 autorefractor; Canon, Tokyo, Japan). Right eyes of all subjects were used for analysis and underwent axial length measurements and OCT imaging. Axial length (AL) measurements were obtained using the IOL master (Carl Zeiss AG, Oberkochen, Germany). Multiple AL measurements (at least 3) were taken and an average value was recorded.

### Spectral domain OCT imaging

Cirrus HD-OCT (Carl Zeiss, Dublin, California, USA) device was used to obtain high-definition images. Macular cube 512×128 and optic disc cube 200×200 were utilized to assess macular and peripapillary RNFL thickness respectively. Macular thickness (average, central,and all subfields) and volume and RNFL thickness (all four quadrants: superior, nasal, inferior and temporal, as well as average) were recorded. Signal strength of 6 or higher was considered acceptable. Internal fixation was used to ensure proper alignment of the eye. All imaging was performed by an experienced ophthalmic photographer or one of the authors. Multiple measurements were taken and the best centered one with good signal strength was chosen for analysis. The macular analysis provided measurements of central subfield thickness (central 1 mm disc) as well as macular thickness in two concentric circles of 3 mm and 6 mm diameters respectively centered at the fovea. The inner and outer circles were in turn divided into four quadrants: superior, nasal, inferior and temporal according to the nine areas A1-9 corresponding to the Early Treatment Diabetic Retinopathy Study (ETDRS Research Group 1985).

### Statistical analysis

Data were entered in a Microsoft Excel sheet, and then transferred to the Statistical Package for Social Sciences program (SPSS, version 19) for data management and analyses. Descriptive statistics were reported as mean and standard deviation, as well as the 5^th^ and 95^th^ percentiles. Distributions of the measurements were graphically presented using histograms. Moreover, correlation between different measurements was done using the Pearson correlation coefficients. Finally, to adjust for the potentially confounding effect of age, gender, and axial length, we carried out multivariate linear regression analyses, where the regression coefficient was reported along with the 95% confidence interval (CI). Paired t-test was used to compare macular thicknesses. P-value <0.05 was considered to indicate statistical significance.

## Results

A total of 113 children and adolescents were enrolled in the study, five subjects were later excluded due to decentered OCT scans. We included 108 patients (mean age 10.7+/−3.1 years), 43 males (mean age 10.7+/−3.4 years) and 65 females (mean age 10.7+/−3.0 years). Data from right eyes of all patients were recorded for analysis. Average spherical equivalent (SE) refraction was −0.02+/−1.77(−4.25 to +5.00) diopters and average axial length was 23.5+/−1.0 (21.5 to 25.8) mm.

**Figure 1:**
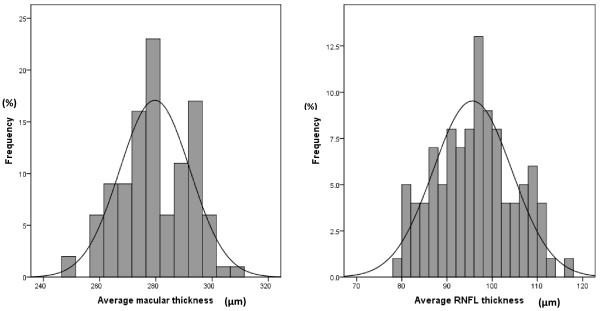
**Normal distribution of average macular thickness (left) and average retinal nerve fiber layer thickness (right).** RNFL, retinal nerve fiber layer.

Mean RNFL and macular thicknesses followed a normal distribution (Figure 1). Patients were divided into four subgroups based on age: 6 up to 9 years of age (n = 35), 9 up to12 years (n = 28), 12 up to15 years (n = 28) and 15 up to18 years (n = 17). Mean RNFL thickness for all patients was 95.6+/−8.7 μm; quadrant thicknesses followed a pattern similar to that in adults with the thickest being the inferior followed by superior then nasal then temporal (Table 1).

**Table 1:** Distribution of retinal nerve fiber layer thickness measurements using Cirrus optical coherence tomography

	**All ages**	**6–9 y**	**9–12 y**	**12–15 y**	**15–18 y**
**Measurements in μm**	**Mean (5**^**th**^**–95**^**th **^**percentile)**	**Mean (5**^**th**^**–95**^**th **^**percentile)**	**Mean (5**^**th**^**–95**^**th **^**percentile)**	**Mean (5**^**th**^**–95**^**th **^**percentile)**	**Mean (5**^**th**^**–95**^**th **^**percentile)**
**Superior**	121 (99–145)	123 (99–151)	118 (96–142)	122 (102–145)	117 (93–131)
**Nasal**	70 (49–94)	70 (49–89)	71 (51–83)	71 (49–95)	69 (43–113)
**Temporal**	66 (54–84)	69 (56–87)	64 (51–83)	68 (57–83)	63 (53–75)
**Inferior**	125 (95–159)	129 (91–163)	122 (101–150)	127 (95–160)	118 (97–131)
**Average**	96 (80–111)	98 (84–111)	93 (80–106)	98 (82–111)	92 (80–101)

y, years.

Mean central macular thickness for all patients measured 249.1+/−20.2 μm, while average macular thickness value was 279.6+/−12.5 μm, and mean macular volume was 10.1+/−0.5 mm^3^ (Table 2). Inner circle values were significantly increased as compared to the outer macular thicknesses (p < 0.0001). There was a significant positive correlation between average RNFL and average macular thickness (r = 0.30, p = 0.002).

**Table 2:** Distribution of macular measurements using Cirrus optical coherence tomography

	**All ages**	**6–9 y**	**9–12 y**	**12–15 y**	**15–18 y**
**Measurements**	**Mean (5**^**th**^**–95**^**th **^**percentile)**	**Mean (5**^**th**^**–95**^**th **^**percentile)**	**Mean (5**^**th**^**–95**^**th **^**percentile)**	**Mean (5**^**th**^**–95**^**th **^**percentile)**	**Mean (5**^**th**^**–95**^**th **^**percentile)**
**Macular Volume, mm**^**3**^	10.1 (9.3–10.7)	10.0 (9.0–10.7)	10.0 (9.3–10.7)	10.2 (9.3–10.8)	10.2 (9.5–11.0)
**Macular thickness, μm**					
Central	249 (219–285)	244 (204–284)	243 (205–262)	255 (223–288)	259 (226–296)
Average	280 (259–298)	277 (250–296)	278 (260–297)	281 (257–304)	283 (263–306)
**Inner Circle**					
Superior	321 (294–348)	315 (288–344)	318 (290–340)	327 (303–356)	329 (301–355)
Nasal	321 (294–347)	315 (291–338)	317 (282–339)	327 (304–358)	329 (308–361)
Temporal	309 (282–337)	304 (276–328)	306 (279–331)	313 (289–342)	314 (281–340)
Inferior	318 (293–343)	313 (290–336)	315 (286–339)	324 (301–350)	321 (295–346)
**Outer Circle**					
Superior	282 (260–308)	283 (256–311)	279 (262–299)	285 (259–321)	284 (288–300)
Nasal	298 (272–322)	295 (271–317)	297 (272–316)	303 (278–328)	300 (272–327)
Temporal	263 (242–287)	261 (237–288)	260 (241–279)	269 (241–339)	265 (243–288)
Inferior	270 (247–295)	268 (245–298)	267 (248–288)	257 (255–301)	268 (246–291)

y, years.

As expected, in the correlation analysis, a significant negative correlation was seen between age and SE(r = −0.33, p = 0.001) and between SE and AL(r = −0.70, p < 0.001). A significant positive correlation was observed between age and AL (r = 0.30, p = 0.02).

On multivariate analysis, RNFL measurements were not affected by gender whereas central macular thickness values were significantly increased in males (p < 0.001). RNFL measurements did not correlate with age but did correlate positively with SE. When controlling for axial length, the correlation with SE remained significant for average RNFL thickness but not for quadrant thicknesses. All macular parameters were consistently positively correlated with age and most were positively correlated with SE (Table 3).When controlling for axial length, correlation with SE was lost and only the positive correlation of the inner circle macular thicknesses with age persisted.

**Table 3:** Multivariate regression analysis of optical coherence tomography parameters by age and spherical equivalent

	**Age**			**Spherical equivalent**		
**Measurements**	**Regression coefficient**	**95% Confidence interval**	**P value**	**Regression coefficient**	**95% Confidence interval**	**P value**
**RNFL thickness**, μm						
Superior	−0.19	−1.08 to 0.7	0.67	2.09	0.53 to 3.65	**0.009**
Nasal	0.47	−0.32 to 1.27	0.24	3.10	1.69 to 4.5	**<0.0001**
Temporal	−0.59	−1.18 to 0.00	0.05	−0.69	−1.74 to 0.35	0.19
Inferior	0.07	−0.97 to 1.12	0.89	5.22	3.37 to 7.06	**<0.0001**
Average	−0.01	−0.51 to 0.49	0.96	2.48	1.59 to 3.37	**<0.0001**
**Macular Volume**, mm^3^	0.03	0.002 to 0.06	**0.03**	0.07	0.02 to 0.12	**0.007**
**Macular Thickness**, μm						
**Central**	1.82	0.64 to 2.99	**0.003**	−0.41	−2.49 to 1.67	0.70
**Average**	0.95	0.16 to 1.74	**0.02**	1.82	0.43 to 3.21	**0.01**
**Outer circle**						
Superior	0.97	0.09 to 1.85	**0.03**	3.11	1.55 to 4.66	**<0.0001**
Nasal	1.06	0.15 to 1.97	**0.02**	1.79	0.18 to 3.39	**0.03**
Temporal	1.23	0.17 to 2.28	**0.02**	1.69	−0.18 to 3.57	0.07
Inferior	0.66	−0.25 to 1.57	0.16	1.77	0.16 to 3.38	**0.03**
**Inner circle**						
Superior	1.85	0.90 to 2.79	**< 0.0001**	1.52	−0.15 to 3.18	0.07
Nasal	1.91	0.95 to 2.86	**< 0.0001**	1.07	−0.61 to 2.74	0.21
Temporal	1.47	0.55 to 2.38	**0.002**	1.19	−0.43 to 2.80	0.15
Inferior	1.40	0.45 to 2.29	**0.003**	0.73	−0.86 to 2.32	0.37

RNFL, retinal nerve fiber layer.

Bolded p values are statistically significant, p < 0.05.

## Discussion

This study reported normative values for RNFL thickness, macular thickness and macular volume in children 6–17 years of age using spectral domain OCT (Cirrus). Correlations with biometric data showed that central macular but not RNFL thickness values were higher in males; additionally, the macular inner circle thickness correlated positively with age and average RNFL thickness correlated positively with SE. Optical coherence tomography, being non-invasive and especially fast, is gaining more popularity in evaluating vitreoretinal diseases of childhood including pediatric glaucoma [20,22]. Spectral domain -OCT, the latest generation of the technology, provides higher resolution and decreased acquisition time, hence is more useful in the pediatric population. Direct comparison of RNFL and macular parameters between TD-OCT and SD-OCT is not possible due to different scanning algorithms [12-14]. Studies using the earlier TD-OCT in children have shown good reproducibility [4,23]. Altemir et al. showed good reliability and repeatability in children using Cirrus SD-OCT [2]. With Stratus OCT, good quality scans could be obtained in 93% and 96% of children [1,3]. Using Spectralis SD-OCT, Turk et al. reported higher feasibility of 99% [16]. We similarly report high quality Cirrus OCT scans in 96% of our enrolled children.

Most available literature reporting normative OCT values in children used TD-OCT [5-11]. Recently, normal values have been reported using Spectralis in Turkish and North American children [16,17] and RTVue-100 OCT in Chinese children [18]. Using Cirrus, Elia et al. reported RNFL but not macular parameters in Caucasian children aged 6–13 years; however, ophthalmologic examination was performed without cycloplegia and OCT parameters were not correlated with biometric data [19]. Our work compliments the latter study by: confirming RNFL normative values on Cirrus, expanding the age group to include 14–17 year-old adolescents, reporting additional normative values for macular thicknesses and volume, and correlating RNFL and macular parameters with age, gender, refractive error and axial length. At the time of our data analysis, we noted a recent report of similar work using Cirrus in another study from Spain; Barrio-Barrio et al. reported RNFL and macular measurements in Caucasian children from three Spanish centers [21]. While Elia and Barrio-Barrio reported normative values in the Spanish population, the current study reported normative Cirrus OCT values in children from the Middle East. The study by Barrio-Barrio tried to address similar objectives as the current study; differences in design include the multicenter nature of their study and the different age group (4–17 years in their study, 6–17 years in our study). Reported normative data in their study were very close to our measurements (Tables 4 & 5), correlations were also in agreement: positive correlation of RNFL with SE and central macular thickness with age and male gender. They similarly excluded subjects with high refractive errors (>5.5 diopters of SE).

**Table 4:** Reported retinal nerve fiber layer thickness measurements by optical coherence tomography in normal children

				Mean RNFL values, μm*				
**OCT**	**Source**	**n**	Age, years*	**Average**	**Superior**	**Nasal**	**Temporal**	**Inferior**
**TD-OCT**								
**OCT 3**	Ahn 2005	72	12.6 ± 2.1	OD106.8 ± 13.0 OS104.3 ± 7.7	OD 132.7 ± 23.9	OD 75.6 ± 13.6	OD 85 ± 14.9	OD 133.3 ± 25.3
					OS 132.7 ± 16.4	OS 63.6 ± 14.1	OS 90.5 ± 20.4	OS 130.9 ± 15.0
**Stratus**	Salchow 2006	92	9.7 ± 2.7	107.0 ± 11.1	135.4 ± 19.3	83.0 ± 18.0	72.5 ± 13.4	136.9 ± 16.9
**Stratus**	Huynh 2006	1369	6.7 ± 0.4	103.7 ± 11.4	129.5 ± 20.6	81.7 ± 19.6	75.7 ± 14.7	127.8 ± 20.5
**Stratus**	El-Dairi 2009	286	8.6 ± 3.1	108.3 ± 9.9	142.9 ± 18.8	83.3 ± 19.2	77.5 ± 15.4	129.4 ± 18.3
**Stratus**	Leung 2010	97	9.7(6.1-17.6)	OD 113.5 ± 9.8 OS 113.1 ± 10.8	OD 146.3 ± 16.3 OS 148.6 ± 19.5	OD 78.3 ± 1 6.1 OS 74.2 ± 14.8	OD 87.3 ± 15.4 OS 86.6 ± 16.6	OD 142.4 ± 1 8.4 OS 143.2 ± 8.7
**Stratus**	Qian 2011	199	10.4 ± 2.7	112.3 ± 9.2	148.7 ± 17.1	74.8 ± 15.0	83.8 ± 13.5	142.1 ± 16.0
**SD-OCT**								
**Spectralis**	Turk 2012	107	10.5 ± 2.9	106.4 ± 9.4	ST 139.0 ± 17.6 SN 102.9 ± 16.0	71.5 ± 10.0	74.3 ± 9.4	IT 144.6 ± 17.2 IN 106.4 ± 19.1
**Spectralis**	Yanni 2012	83	8.9 (5–15)	107.6 ± 1.2	ST 145.1 ± 2.2 SN 116.2 ± 2.8	84.5 ± 1.9	76.5 ± 1.9	IT 147.0 ± 2.1 IN 125.4 ± 3.0
**RTVue-100**	Tsai 2012	470	9.2 (6.5-12.5)	109.4 ± 10.0	133.9 ± 18.1	71.1 ± 11.3	90.4 ± 14.3	142.2 ± 19.5
**Cirrus**	Elia 2012	344	9.2 ± 1.7	98.5 ± 10.8	123.6 ± 19.5	71.3 ± 13.5	69.4 ± 11.3	130.2 ± 18.1
**Cirrus**	Barrio-Barrio 2013	283	9.6 ± 3.1	97.4 ± 9.0	124.7	69.7	67.4	128.0
**Cirrus**	Al-Haddad 2013 current study	108	10.7 ± 3.1	95.6 ± 8.7	120.6 ± 13.8	70.1 ± 13.0	66.4 ± 8.9	124.8 ± 18.1

*mean ± standard deviation or mean (range).

ST, supero-temporal; SN, supero-nasal; IT, infero-temporal; IN, infero-nasal.

**Table 5:** Reported macular thickness measurements by optical coherence tomography in normal children

				Macular parameters*		
**OCT**	**Source**	**n**	Age, years^*^	**Volume, mm**^**3**^	**Average thickness, μm**	**Central thickness, μm**
**TD-OCT**						
**Stratus**	Huynh 2006	1543	6.7 ± 0.4	6.9 ± 0.4	Inner: 264.3 ± 15.2 Outer: 236.9 ± 13.6	193.6 ± 17.9
**Stratus**	El-Dairi 2009	286	8.6 ± 3.1	6.9 ± 0.3	Inner: 268.3 ± 13.6 Outer: 240.0 ± 12.8	188.8 ± 25.0
**Stratus**	Eriksson 2009	56	10.1 (5–16)	7.1 ± 0.3	Inner: 279 ± 13 Outer: 245 ± 12	204 ± 19
**SD-OCT**						
**Spectralis**	Turk 2012	107	10.5 ± 2.9	0.26 ± 0.01	326.4 ± 14.2	211.4 ± 12.2
**Cirrus**	Barrio-Barrio 2013	281	9.6 ± 3.12	10.2 ± 0.5	283.6 ± 14.1	253.9 ± 19.8
**Cirrus**	Al-Haddad 2013 current study	108	10.7 ± 3.14	10.1 ± 0.5	279.6 ± 12.5	249.1 ± 20.2

*mean ± standard deviation or mean (range).

Tables 4 and 5 compare our OCT values with earlier literature. Average RNFL thickness in this study measured 96 μm comparable to others using Cirrus (97-98 μm) but lower than values recorded using Stratus (104-113 μm), Spectralis (106-107 μm) and RTVue (109 μm). The quadrant thickness distribution followed the classic “double hump” pattern seen in adults with the thickest being the inferior followed by the superior then nasal and temporal, consistent with the “ISNT rule”. However, a number of reports have shown exceptions to this rule [3,5,7,9,18].

Macular parameters show the greatest variability among different OCT devices and algorithms [14]; discrepancies have also been noted across different versions of OCT [15,24]. Few studies have reported macular parameters in normal children (Table 5). Mean macular volume in our study was 10.1 mm^3^ similar to other studies using Cirrus but much higher than those obtained by Stratus (7 mm^3^) or Spectralis (0.25 mm^3^); this also applied to central macular thickness values. Average macular thickness was highest on Spectralis (326 μm), followed by Cirrus (280-284 μm) and Stratus (240-270 μm). Yanni et al. reported mean macular thickness (271 μm) on Spectralis but proceeded to segment the retinal layers and perform normative segmentation values, hence their data were not included in Table 5[17]. In the current study, inner macular thicknesses were statistically significantly higher than outer macular values in all quadrants similar to results from Stratus [4,5].

Discrepancies noted in recorded normative OCT values could also be related to confounding variables like ethnicity, race, gender, age, SE and AL measurements. All our subjects were white and Middle Eastern while the other two reports using Cirrus came from a Spanish population. Gender differences applied only in central macular thickness measurements which were significantly increased in males. This finding is in agreement with Barrio-Barrio [21] and Huynh et al. [8]. Gender differences may need to be accounted for during OCT interpretations.

In this study, RNFL values were not affected by age in agreement with other reports [5,9,16,21]. Some authors suggested that nerve fiber layer losses happened later in life after the age of 50 years, hence the absence of RNFL correlation with age in children [25]. By contrast, we found a strong positive correlation between age and macular parameters, similar to the findings by Barrio-Barrio et al. [21] and Huynh et al. [8].

High refractive errors may affect OCT measurements; therefore, we excluded patients with SE more than 7 diopters. Still we noted a strong persistent positive correlation between SE and average RNFL thickness measurements after controlling for axial length. Many authors have observed positive correlations with SE [3,8,11,18]. Tsai et al. reported an increase in average RNFL thickness of 1.7 μm for every diopter of hyperopia [18]. On the other hand, Turk et al. did not detect any correlations with SE or axial length [16].

Strengths of this study include the large age range (6–17 years) of enrolled children, the use of the new generation Cirrus SD-OCT, the completion of a comprehensive eye examination (with cycloplegic retinoscopy) by a pediatric ophthalmologist, the recording of both normative RNFL and macular parameters, and the detailed biometric correlations. Limitations of this work include the mostly uniform ethnic group (white and Middle Eastern) so the effect of race and ethnicity could not be tested. We also excluded patients with high refractive errors and increased cup to disc ratios; normative data for these groups were not established. Additionally, our study was hospital-based and not population-based. However, patients in this setting received a comprehensive examination, and biometric data were recorded.

## Conclusions

This study established normal reference ranges for RNFL and macular parameters measured by Cirrus SD-OCT in healthy Middle Eastern children 6–17 years of age. This adds another database to the available literature on normative values using other OCT devices and facilitates evaluation of OCT measurements in children with optic neuropathies, glaucoma and macular diseases. The data presented are for white Middle Eastern children; hence, other races and ethnicities should be studied in future research. Variability with age and gender warrants special consideration during OCT interpretations.

## Competing interests

The authors declare that they have no competing interests.

## Authors’ contributions

CA conceived and designed the study and drafted the manuscript. AB participated in enrolling patients, analyzing results and drafting the manuscript. MJ enrolled patients, analyzed results and drafted the manuscript. VM enrolled patients and analyzed results and drafted the manuscript. HT participated in study design,statistical analysis and drafting the manuscript. BN participated in study conception, design and analysis of results. All authors reviewed the final manuscript critically and approved it. All authors read and approved the final manuscript.

## Pre-publication history

The pre-publication history for this paper can be accessed here:

http://www.biomedcentral.com/1471-2415/14/53/prepub
